# Maternal determinants of dietary patterns in infancy and early childhood in the *Growing up in New Zealand* cohort

**DOI:** 10.1038/s41598-023-49986-2

**Published:** 2023-12-20

**Authors:** Teresa Gontijo de Castro, Amy Lovell, Leonardo Pozza Santos, Beatrix Jones, Clare Wall

**Affiliations:** 1 Department of Nutrition and Dietetics, Faculty of Health and Medical Sciences, University of Auckland, Grafton Road - Bldg 219, Level B, Room B01, Auckland, 1010 New Zealand; 2 Department of Epidemiology and Biostatistics, School of Population Health, University of Auckland, Auckland, New Zealand; 3 Department of Nutrition, Federal University of Pelotas, Pelotas, Brazil; 4 Department of Statistics, Faculty of Sciences, University of Auckland, Auckland, New Zealand

**Keywords:** Epidemiology, Paediatric research

## Abstract

Given the importance of diet in early life, assessing children’s diet is crucial to guide interventions. Using data from a nationally generalizable New Zealand (NZ) birth cohort we examined children’s dietary patterns at 9- (n = 6259), 24- (n = 6292), and 54-months (n = 6131), and their association with maternal sociodemographic and health behaviours. At each time-point, children's dietary patterns were identified using principal components analysis. We then used multivariate linear regression to examine associations between each pattern and maternal variables. At 9-, 24- and 54-months, two dietary patterns were identified, explaining 36.4%, 35.3% and 33.6% of children's intake variability, respectively. *Refined high in sugar, salt and fat* dietary pattern, at all time-points, was characterized by high positive loadings in white/refined breads and cereals, and items with high sugar, sodium, and fat content. At 24-months, *Refined high in sugar, salt and fat* also included a high positive loading with protein food groups. *Fruit and vegetables* dietary pattern, at all time-points, had high positive loadings for fruits and vegetables (with type varying across time-points). *Fruit and vegetables* also included high loading in whole grain options of breads and cereals at 24-months and the protein food group was part of this dietary pattern at 9- and 54-months. Children’s scores on the *Refined high in sugar, salt and fat* pattern had strong associations with maternal smoking habits, education level, ethnicity, and maternal scores in the “*Junk*” and “*Traditional/White bread*” dietary patterns (constructed from an antenatal interview). Children’s scores on the *Fruit and vegetables* pattern had strong associations with the maternal scores in the dietary pattern *“Health Conscious”*. Interventions to improve diet in early life in NZ need to be responsive to ethnicity and suitable for people of all education levels. Interventions that improve maternal health behaviours may also improve children’s diet.

## Introduction

Feeding practices in early life including optimal breastfeeding duration, timely introduction of foods, and intake of healthy foods are associated with higher diet quality in childhood and better short- and long-term health outcomes such as the decreased risk of developing obesity^1–13^. Several determinants influence feeding practices in the first years of life, with maternal health behaviours and sociodemographic inequalities playing an important role^8,13–29^.

Intakes of many foods and nutrients are strongly correlated, making it difficult to isolate the effect (s) of a specific food or nutrient. This is where dietary patterns analyses can assist, as they make use of the correlated nature of dietary data to identify patterns of total food consumption^11^. Dietary pattern analyses represent a holistic assessment of diets which summarises the combined and potentially synergistic effects of different foods that contribute to the usual dietary intake of a population ^9,11,30,31^. For this reason, dietary patterns have been identified as a more realistic representation of dietary habits, as it can be translated into guidelines for the public more easily^9,11^, and are useful for understanding the associations between diet quality and health outcomes, including childhood obesity^9,11,26,30,32–34^. Previous studies have showed that dietary patterns energy-dense, high-sugar, high-fat, and low-fibre foods appear to be established in the early years of life and tend to track throughout the life course^8,10,22,31,33,35,36^. Therefore, this period of life represents a relevant window of opportunity for promoting dietary trajectories which have a positive and prospective impact on health and wellbeing^2,11,33,35^.

Childhood overweight/obesity is a public health problem in New Zealand (NZ), which has great ethnic and socioeconomic disparities^37^. NZ was placed second among the countries of the Organisation for Economic Cooperation and Development/European Union for the prevalence of overweight/ obesity in 5–19-year-olds (39.5%)^38^. Despite the importance of early feeding practices and their reported associations with child obesity^9–13^, NZ has limited nationally representative or generalizable information on children's diet quality, especially among children under five. Information is only available from a national child nutrition survey conducted two decades ago, which involved children aged 5–14 years^39^. Recently, information from the *Growing up in NZ* cohort study provided the first nationally generalizable information on breastfeeding indicators, timing of food introduction, and diet quality in the first 5 years of life (which used dietary indexes measuring adherence to national Food and Nutrition Guidelines)^14–17^. In this study, we used the *Growing up in NZ* cohort data to provide nationally generalizable information on (*i)* dietary patterns at three time-points in early life (9-, 24- and 54-months) and; (*ii)* determine associations between the identified dietary patterns and maternal health behaviours and sociodemographic characteristics. Findings from this study aim to guide interventions designed to improve and narrow inequities in diet quality in early life in NZ and, subsequently, to positively impact health and wellbeing indicators of future generations.

## Methods

### Data collection and population

This cross-sectional study used data from *Growing Up in New Zealand*, a contemporary NZ birth cohort which enrolled 6,822 pregnant women and their 6,853 children who survived to age 6 weeks^40^. The eligibility of a pregnant woman was determined by an estimated delivery date between 25 April 2009 and 25 March 2010, and residence in the region of NZ defined by boundaries of three contiguous district health boards. At birth, the cohort was broadly generalizable to the NZ births that took place from 2007 to 2010, allowing for robust analyses by ethnicity and socioeconomic position^40^.

This study used information from five data collection waves (antenatal, six weeks after birth, and at 9-, 24-, 31-, and 54-months). Information on maternal sociodemographic and health behaviour characteristics were obtained from the antenatal, 24- and 54- face-to-face computer-assisted personal interviews (CAPIs). Child perinatal information (sex, singleton/twin-triplet, birth weight and gestational age) was obtained from the six-week computer-assisted telephone interview (CATI). Information on children's dietary intake was obtained from the 9-, 24- and 54-month children’s CAPIs. Duration of any breastfeeding was estimated from data obtained at the six-week CATI and at the 9-month and 31-month CAPIs^14^.

**Figure 1:**
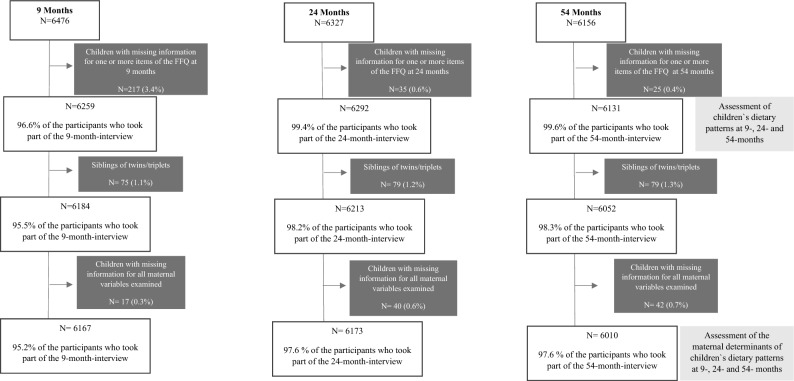
Flow-chart showing the number (%) of children included in the analyses at 9-, 24- and 54- months.

Of the 6,853 children enrolled in the cohort, the respective proportions of children for whom the 9, 24-, and 54-month interviews were completed were 92% (n = 6237), 90% (n = 6156) and 89.8% (n = 6156)^41^. To describe children's dietary patterns, those with no or incomplete information on dietary intake were excluded: 3.4% (n = 217), 0.6% (n = 35) and 0.4% (n = 25) of the children who took part of the 9-, 24-and 54-month interviews, respectively. Thus, dietary patterns were described for 96.6% (n = 6259), 99.4% (6292) and 99.6% (6131) of the children at the 9-, 24-and 54-month interviews, respectively. To examine associations between the children's scores for the dietary patterns at each time-point and maternal sociodemographic and health behaviour characteristics we excluded: (*i)* siblings of twins and two siblings of triplets: 1.1% (n = 75; 9-months); 1.2% (n = 79; 2- months) and 1.3% (n = 79; 54-months) and; (*ii)* children with missing information for all the maternal covariates examined: 0.3% (n = 17; at 9-months); 0.6% (n = 40; at 24-months) and 0.7% (n = 42; at 54-months). The analyses of associations included 95.2% (n = 6167), 97.6% (6173) and 97.6% (n = 6010) of the children who took part of the 9-, 24-and 54-month interviews, respectively (Fig. 1). Twins/triplets were included when identifying the dietary patterns because their dietary patterns will be used in future analyses within the *Growing Up in New Zealand* cohort study. We randomly excluded siblings of twins and two siblings of triplets from the analyses of associations to guarantee only the inclusion of independent observations.

### Dietary patterns at 9, 24 and 54 months

*Growing Up in New Zealand* used semi-quantitative food frequency questionnaires (FFQs) to assess infant and children’s dietary intakes at the 9-, 24- and 54-month interviews. At the 9-month interview, a 25-food items FFQ was used, where mothers were asked to report their infants’ usual frequency of intake of foods and drinks^15^. At the 24- and 54-month interviews, a  62-food items FFQ was used; mothers were asked to report their children’s usual intake in the previous four weeks as well as the amount typically eaten, relative to a “standard” serving (two or more, one, ½ and ¼ servings). The only difference in the FFQs used at 24- and 54-month interviews was that “toddler formula milk” and “toddler prepared meals” were part of the 24-month FFQ but not of the 54-month FFQ. Similarly, “avocados” and “crackers” were part of the 54-month-FFQ but not of the 24-month-FFQ^16^.

The FFQs were administered to children's caregivers during the CAPI interviews at 9-, 24- and 54-months and show-cards containing the photos of usual servings of each food/food grouping listed in the FFQs were shown to help the caregivers^16^. *Growing Up in New Zealand* FFQs were designed to measure the cohort’s adherence to nationally recommended intakes of food groups for infants and preschoolers at the time of the interviews^42,43^, rather than to provide a full portrait of children’s usual intakes. To guarantee the inclusion of the foods commonly consumed by NZ infants and preschoolers, *Growing Up in New Zealand* FFQs’ food lists were revised based on studies conducted in NZ: an investigation conducted in Auckland-NZ with 6- to 23-month-olds^44^ and a nationally representative survey on diets for 5-to-14-year-olds^39^. We have previously created dietary indexes measuring *Growing Up in New Zealand* adherence to National Food and Nutritional Guidelines at 9-,24- and 54-months. These indexes were created using data from the FFQs administered at these time-points and the indexes convergent construct validity was demonstrated by their ability to discriminate the cohort's sociodemographic characteristics^15,16^.

For examining children's dietary patterns at 9-, 24- and 54-months, we grouped the FFQs' foods and food groups based on the national recommendation of food and food groups for infants and preschoolers ^42,43^. These food groups/items were grouped by adding the daily frequency of intake of foods at 9-months and the daily number of servings at 24- and 54-months. Thus, at 9-months food items/groups were grouped under 10 foods groups: Vegetables; Fruit; Breads and Cereals; Baby foods-iron enriched; Suitable infant formula milk; Other milk products; Protein Group: Meat, chicken or meat dishes, Fish or fish dishes (fresh and canned), Shellfish, Eggs, Soyfoods, Tofu, Soy desserts, Nuts or peanut butter; Inappropriate drinks—not recommended to infants; Inappropriate foods—high content of added sugars; Inappropriate foods—high content of sodium and fat. Similarly, at 24- and 54-months the food items/groups were grouped under 13 food groups: Green leafy vegetables; Other types of vegetables; Citrus fruit; Other types of fruit; Breads and cereals-white, refined options; Breads and cereals-brown, wholegrain options; Milk, milk products or suitable alternatives- whole milk, non-reduced fat options; Milk and milk products- skim/trim milk, low-fat options; Protein Group: Lean meat, poultry, fish, shellfish, eggs, legumes, nuts and seeds; Processed meats; Inappropriate drinks—high content of added sugars; Inappropriate foods—high content of added sugars; Inappropriate foods—high content of sodium and fat, especially saturated fat. At 24 months we also included toddler formula milk (further details on Table S1).

### Mother and child covariates

The selection of maternal sociodemographic and health behaviour characteristics examined in this study was based on prior literature demonstrating their associations with children’s dietary patterns^13–21,23^. We also adjusted in the models examining the associations between maternal variables and children's dietary patterns the following children's covariates described in the literature as associated with diet quality in childhood: exact age at the time of 9-,24- and 54-month interviews (in months), sex (male/female), gestational age (in weeks-continuous), birth weight (in grams-continuous), and any breastfeeding duration (never breastfed/< 6 months/ ≥ 6 months)^4,6,8,15,16,20–23,28^ .

The following antenatal maternal sociodemographic and health behaviour variables were examined: level of education completed, age, ethnicity, body-mass-index, physical activity before/during pregnancy, and maternal dietary patterns. Maternal smoking habits were examined at the three time-points. Before and during pregnancy smoking patterns were categorized as: continued smoking, stopped smoking during pregnancy and non-smokers. At the 24- and 54-month interviews, maternal cigarette smoking was assessed by asking the mothers if they were currently smoking regularly at least one cigarette/daily (yes/no). A NZ index of deprivation (NZDep) measured antenatally and at the 24-month and 54-month interviews was used. Children’s home addresses were geocoded and linked to the NZDep (in deciles) which are also used as the small area measure of neighbourhood deprivation^45,46^. The NZDep2006 was used at the 9- and 24-month interviews while the NZDep2013 was used at the 54-month interview. The NZDep2006 combines nine socioeconomic characteristics from the 2006 census data collected at aggregations of approximately 100 people and assigned to individual households based on geo-coded address data^45^. The NZDep2013 is derived similarly to NZDep2006, however, using 2013 census data^46^. Maternal ethnicity was prioritised using the Statistics NZ classification Level 1: Māori, Pacific Peoples, Asian, Middle Eastern, Latin American, and African (MELAA), Other, and European^47^. MELAA and Other were combined for analysis purposes. Maternal pre-pregnancy BMI was calculated based on self-reported weight and height and classified according to the World Health Organization (WHO) cut-offs^48^. Physical Activity (PA) before and/or during pregnancy was estimated using the International Physical Activity Questionnaire (IPAQ). Women who engaged in moderate PA for at least 30 min for at least 5 out of 7 days, or vigorous PA for at least 30 min on at least 2 out of 7 days were classified as participating in moderate/vigorous activity^49^ and this variable was categorized as: no moderate/vigorous PA before or during pregnancy, moderate/vigorous PA before and during pregnancy, and moderate/vigorous PA only before or only during pregnancy.

Maternal diet during pregnancy was assessed using a semi-quantitative 44-item FFQ. The FFQ's food list was developed based on the New Zealand Ministry of Health guidelines for healthy pregnant women^50^ and food intake was recalled over the 4 weeks preceding the antenatal interview (with frequencies of intake ranging from not at all, or less than once a month, to a number of times per month, week, or day). Show-cards containing photos of usual servings of food/food grouping listed were shown to participants^51^. A previous NZ study identified four distinct dietary patterns (DPs) during pregnancy that explained 23.4% of the total variance in dietary intake. High positive loadings for confectionary, snacks, takeaways, hot chips, soft/energy drinks, cakes/biscuits, processed meats, battered fried fish/seafood and ice cream characterized the “*Junk”* dietary pattern while the “*Healthy Conscious”* dietary pattern had high positive loadings for vegetables, brown whole meal bread, cheese, non-citrus fruits, yoghurt, dried fruits, high fibre cereal and Vegemite/Marmite. The “*Traditional/White”* dietary pattern had high positive loadings for whole/standard milk, white bread, margarine, jam/honey/marmalade, peanut butter/Nutella, and a high negative loading for skim/trim milk. High positive loadings for noodles/rice/pasta, seafood, chicken, green leafy vegetables, eggs, red meat, processed fish, and citrus fruits characterized the *“Fusion”* dietary pattern^51^.

### Statistical analyses

Children's dietary patterns at the 9-,24-and 54-month interviews were identified using principal components analyses. Food/food groups were entered in the analyses as daily frequency of intake (9-months) and daily serving’s intake (24- and 54-months), as previously described, and were standardized using the correlation matrix. Rotated factor solutions ranging from 2 to 4 were generated before deciding which factors to retain. The numbers of patterns (components) identified at each time-point were based on factors with components with eigenvalues > 1.5, breaks in the scree plots and the interpretability and meaningfulness of the matrix after varimax rotation. We considered that food items had moderate to large correlations with the identified dietary patterns adopting the threshold of > 0.45 or <  − 0.45 for the factor loadings. For each dietary pattern, naming was based on food groupings with high positive factor loadings.

Associations between the children’s factor scores for each dietary pattern identified at 9-, 24-and 54-month interviews (dependent variables) and the maternal socio-demographic and health behaviour characteristics (independent variables) were examined using multivariate linear regression models with associations described using *β*-coefficients and 95% confidence intervals (95% CI). At the three time-points, models were adjusted for children's sex and age. The 9-months models were also adjusted for children’s birth weight, gestational age, and duration of any breastfeeding. Analyses of covariance (unadjusted and adjusted) were also performed between the dependent variables and the independent variables with 2 or more categories, to get an omnibus test of the importance of that variable. All analyses were performed using SPSS software (version 25, IBM SPSS Statistics).

### Ethics approval and consent to participate in the study

*Growing up in New Zealand* was conducted according to the guidelines of the Declaration of Helsinki and all procedures involving human subjects were approved by the Ministry of Health’s Northern Y Regional Health and Disability Ethics Committee (NTY/08/06/055). Written informed consent was obtained from all children's mothers/caregivers^40^.

## Results

### Population

Table S2 presents the characteristics of the children and their mothers included in the analyses of dietary patterns at the 9-, 24-and 54-month interviews. The mean (SD) child’s age at the 9-, 24- and 54-month interviews were, 9.0 (0.9), 24.3 (2.0) and 54.0 (1.6) months, respectively. Most children were singletons and males represented 51.6%, 51.6% and 51.5% of the children at the 9-, 24- and 54-month interviews. Among the children included in the analyses at 9-months, approximately one-third were not breastfed or were breastfed for less than 6 months, had a mean (SD) of 3490 (583.6) grams for birth weight and 39.1 (1.9) weeks for gestational age. At all time-points, approximately 4 in 10 mothers had education level higher or equal bachelor’s degree and approximately 6% had no secondary school qualification. At the 9-, 24- and 54-month interviews, 37%, 35.9% and 33.2% of the children belonged to the most deprived neighbourhoods (deciles 8–10 of the NZDep), respectively. Approximately half of the mothers were of European ethnicity, most were aged 20–34 years and approximately 4 in 10 mothers had a pre-pregnancy BMI ≥ 25 kg/m^2^. Approximately 3 in 10 mothers reported practicing moderate/vigorous PA before and during pregnancy. Antenatally, 19.3% of mothers reported smoking before and during pregnancy and respectively at the 24- and 54-month interviews 13.4% and 13.1% of the mothers were smoking at least 1 cigarette/day.

### Dietary patterns at 9-, 24- and 54-months

At each of the interviews, two distinct dietary patterns were identified and together explained 36.4%, 35.3% and 33.6% of the food intake variance at 9-, 24- and 54-months, respectively (scree plots are illustrated in Figure S1). At each time-point the dietary patterns were named based on the food items with higher factor loadings. The first dietary pattern was named *Refined high in sugar, salt, and fat*, and was characterized, at all time-points, by high positive loadings in white/refined breads and cereals and items with high content of sugar, sodium, and fat. At 24-months, *Refined high in sugar, salt, and fat* also had high positive loading in the protein group (which included lean meats, poultry, fish, shellfish, eggs, legumes, nuts, and seeds). The second dietary pattern was named *Fruit and vegetables* and at all time-points had high positive loadings for fruits and vegetables (with type varying at 9-, 24- and 54-months). *Fruit and vegetables* at 24- months had also high positive loadings for whole grain breads and cereals. At 9- and 54-month, *Fruit and vegetables* had also positive loading in the protein group (at 9-months this group included Meat, chicken, or meat dishes; Fish or fish dishes (fresh and canned); Shellfish; Eggs; Soyfoods-tofu, soy deserts, Nuts/peanut butter; at 54-months this group included lean meats, poultry, fish, shellfish, eggs, legumes, nuts and seeds) (Table 1).

**Table 1:** Factor loadings of the food items and groups in the 2 dietary components obtained using principal component analysis at 9- (n = 6259), 24- (n = 6292) and 54-months (n = 6131).

Food items or groups	Refined high in sugar sodium and fat	Fruit and vegetables
**9-month interview-** Frequency of intake (day)		
***Variance explained (%)***	***19.28***	***17.11***
Vegetables (raw or cooked)	−.081	**.777**
Fruit (fresh or canned)	−.015	**.790**
Protein group: Meat, chicken, or meat dishes; Fish or fish dishes (fresh and canned); Shellfish; Eggs; Soyfoods-tofu, soy deserts, Nuts/peanut butter	.393	**.482**
Breads/toast; Other cereal	**.533**	.287
Baby foods (iron-enriched): Baby breakfast cereal; Baby rice; Rusks	−.305	.152
Infants' suitable formula milks	.064	−.245
Milk puddings, rice pudding, yoghurt, custards	.354	.217
Inappropriate foods-high content of added sugars	**.715**	−.024
Inappropriate foods-high content of sodium and fat	**.694**	−.165
Inappropriate drinks-not recommended to infants	**.517**	−.099
**24-month interview-** Serves (day)		
***Variance explained (%)***	***23.27***	***12.00***
Vegetables (green leafy)	.368	.202
Vegetables (other types)	.376	**.539**
Fruit (citrus)	.408	.227
Fruit (other types)	.431	**.483**
Breads and cereals-white, refined options	**.682**	−.307
Breads and cereal-brown, wholegrain options	−.100	**.713**
Milk and milk products- whole milk, non-reduced fat options	.254	.431
Milk and milk products- skim/trim milk, low-fat options	−.036	.142
Toddler formula milk	−.010	−.360
Protein group: Lean meats, poultry, fish, shellfish, eggs, legumes, nuts, and seeds	**.606**	.283
Processed meats-high content of sodium and fat	**.476**	.178
Drinks-high content of added sugars	**.675**	−.074
Foods-high content of added sugars	**.657**	−.102
Foods-high content of sodium and fat	**.779**	−.007
**54-month interview-** Serves (day)		
***Variance explained (%)***	***18.18***	***15.42***
Vegetables (green leafy)	−.099	**.623**
Vegetables (other types)	.016	**.744**
Fruit (citrus)	.166	.399
Fruit (other types)	.154	**.616**
Breads and cereals-white, refined options	**.620**	.183
Breads and cereal-brown, wholegrain options	−.055	.282
Milk and milk products- whole milk, non-reduced fat options	.238	.387
Milk and milk products- skim/trim milk, low-fat options	.012	.020
Protein group: Lean meats, poultry, fish, shellfish, eggs, legumes, nuts, and seeds	.344	**.481**
Processed meats- high content of sodium and fat	**.515**	.155
Drinks-high content of added sugars	**.651**	.004
Foods-high content of added sugars	**.700**	-.015
Foods-high content of sodium and fat	**.749**	.075

Loadings above 0.45 were bold.

### Maternal covariates associated with children's dietary patterns at 9-, 24- and 54-months

The unadjusted associations between the children’s scores in the two dietary patterns at each interview and the maternal socio-demographic and health behaviour characteristics are presented in Supplementary Tables S3–S5.

In the adjusted models, for all time-points, lower maternal educational attainment was significantly associated with children's higher scores in the *Refined high in sugar, salt, and fat* dietary pattern. The largest difference was between mothers with a bachelor’s degree or higher, and those who had not completed a secondary qualification, with intermediate scores for secondary or trade qualifications. For all time-points, compared to children living in the least deprived neighbourhoods (deciles 1–3), those in the most deprived deciles (8–10) scored higher in the *Refined high in sugar, salt, and fat* pattern. Maternal ethnicity was also significantly associated with the *Refined high in sugar, salt, and fat* pattern at all time-points. In comparison to children from European mothers, children scored higher in the pattern if their mothers: were from Māori ethnicity (at 9-, 24- and 54-months), or Asian and Pacific ethnicities (at 24- and 54-months). Maternal age at pregnancy influenced children's score in the *Refined high in sugar, salt, and fat* pattern at 9- and 24-months, but this influence was attenuated at 54-months. Children scored higher in this pattern if their mothers were younger than 35 years of age (compared to children from mothers aged ≥ 35 years). At all time-points, children scored higher in the *Refined high in sugar, salt, and fat* pattern if their mothers were smokers and if their mothers had high antenatal scores in the “*Junk*” and “*Traditional/White”* dietary patterns. Conversely, children of mothers with high scores in the “*Health conscious”* dietary pattern scored lower in the *Refined high in sugar, salt, and fat* at all time-points (Tables 2, 3, 4). Children whose mothers had antenatal high score in the *“Fusion”* dietary pattern scored higher in the *Refined high in sugar, salt, and fat* pattern at 24-months (Table 3) but scored lower in this dietary pattern at 9-months (Table 2).

**Table 2:** Adjusted associations between the children's dietary patterns at the 9-month-interview and antenatal maternal sociodemographic and health behaviour characteristics.

Covariates	N (%)	Refined high in sugar sodium and fat N = 5054; Adjusted R^2^ = 0.21^#^				Fruit and vegetables N = 5055; Adjusted R^2^ = 0.17^#^			
		Adjusted β^†^	95% CI	*P* value*	*P* value**	Adjusted β^†^	95% CI	*P* value*	*P* value**
**Antenatal maternal characteristics**									
***Highest level of education***									
Bachelor’s degree or higher	2413 (39.5)	*Ref*			0.005	*Ref*			< 0.001
Diploma/Trade cert/NCEA 5–6	1881 (30.8)	0.047	−0.012; 0.106	0.117		−0.262	−0.326; −0.198	< 0.001	
Secondary school/NCEA 1–4	1416 (23.2)	0.014	−0.053;0.080	0.687		−0.279	−0.351; −0.207	< 0.001	
No secondary school qualification	396 (6.5)	0.209	0.091;0.327	0.001		−0.336	−0.464; −0.208	< 0.001	
***Prioritised ethnicity***									
European	3041 (49.7)	*Ref*				*Ref*			< 0.001
Māori	1101 (18.0)	0.177	0.105; 0.249	< 0.001	0.004	−0.257	−0.335; −0.179	< 0.001	
Pacific	849 (13.9)	0.078	−0.010;0.167	0.084		−0.220	−0.316; −0.124	< 0.001	
Asian	919 (15.0)	−0.023	−0.104;0.058	0.572		−0.286	−0.373; −0.198	< 0.001	
MELAA and others	206 (3.4)	0.094	−0.032; 0.221	0.144		0.002	−0.136; 0.139	0.983	
***Age group***									
> 35 years	1568 (25.6)	*Ref*			< 0.001	*Ref*			0.010
20–34 years	4277 (69.9)	0.097	0.041; 0.152	0.001		−0.058	−0.118; 0.002	0.056	
< 20 years	277 (4.5)	0.435	0.306; 0.565	< 0.001		−0.131	−0.272; 0.009	0.067	
***Neighbourhood deprivation (NZDep2006)***									
1–3 Least deprived	1567 925.6)	*Ref*			0.097	*Ref*			< 0.001
4–7	2281 (37.3)	0.007	−0.052; 0.066	0.820		−0.018	−0.082; 0.046	0.577	
8–10 Most deprived	2272 (37.1)	0.093	0.026; 0.160	0.007		−0.146	−0.219; −0.074	< 0.001	
**BMI (kg/m**^**2**^**)**									
< 25.0	3208 (59.2)	*Ref*			0.010	*Ref*			0.178
25–29.99	1220 (22.5)	0.004	−0.055; 0.064	0.885		−0.054	−0.118; 0.011	0.102	
> 30.0	993 (18.3)	0.024	−0.045; 0.092	0.494		−0.003	−0.077; 0.071	0.940	
**Physical activity before and during pregnancy**									
Moderate/vigorous physical activity before and during pregnancy	1868 (33.8)	*Ref*			0.085	*Ref*			0.832
Moderate/vigorous physical activity only before or during pregnancy	1469 (26.6)	−0.027	−0.087; 0.033	0.375		0.022	−0.043; 0.087	0.509	
No Moderate/vigorous physical activity before and during pregnancy	2193 (39.6)	−0.030	−0.086; 0.025	0.287		−0.009	−0.069; 0.051	0.779	
***Smoking before and during pregnancy***									
Non-smokers before and during pregnancy	4444 (80.6)	*Ref*			< 0.001	*Ref*			0.316
Stopped smoking during pregnancy	537 (9.7)	−0.038	−0.121; 0.045	0.371		−0.068	−0.158; 0.022	0.136	
Smokers before and during pregnancy	532 (9.6)	0.316	0.222; 0.410	< 0.001		−0.012	−0.114; 0.090	0.814	
***“Junk” dietary pattern score***	5115 (100.0)	0.134	0.109; 0.160	< 0.001	–	−0.032	−0.059; −0.004	0.027	–
***“Health Conscious” dietary pattern score***	5115 (100.0)	−0.035	−0.061; −0.009	0.008	–	0.142	0.114; 0.171	< 0.001	–
***“Traditional/White” dietary pattern score***	5115 (100.0)	0.133	0.105; 0.161	< 0.001	–	−0.073	−0.103; −0.043	< 0.001	–
***“Fusion” dietary pattern score***	5115 (100.0)	0.019	−0.007; 0.044	0.162	–	0.072	0.044; 0.100	< 0.001	–

*Adjusted β* adjusted beta-coefficient, *Adjusted R*^*2*^ adjusted r-square of the model, *CI* confidence interval, *NCEA* National Certificate of Educational Achievement, *Ref* category of reference, *BMI* body mass index, *NZDep2006* neighbourhood deprivation index 2006.

^**#**^Models adjusted by child sex (female/male) and exact age at the 9-month interview in months (continuous), gestational age in weeks (continuous), birth weight (continuous) and breastfeeding duration (never breastfed/< 6 months/≥ 6 months).

Missing: models at 9 months (n): Child’s sex (18); child age at the 9-month interview (^¶^); gestational age (24); breastfeeding duration (14); maternal education (61); maternal ethnicity (51); maternal age; (45); neighbourhood deprivation index (47); maternal smoking patterns (654); maternal dietary patterns-Junk, Health Conscious, Traditional/White and Fusion (1052).

*t-test, ** F-test.

^†^Average increase or decrease in the dietary patterns loading scores in relation to the categories of reference.

^¶^As per *Growing up in New Zealand* study anonymity requirement, “< 10” represents greater than zero and less than 10 children in the cell.

**Table 3:** Adjusted associations between the children's dietary patterns at the 24-month-interview and maternal sociodemographic and health behaviour characteristics (antenatally and at the 24-month-interview).

Covariates	N (%)	Refined high in sugar sodium and fat N = 3818; Adjusted R^2^ = 0.22^#^				Fruit and vegetables N = 3822; Adjusted R^2^ = 0.10^#^			
		Adjusted β^†^	95% CI	*P* value*	*P* value**	Adjusted β^†^	95% CI	*P* value*	*P* value**
**Antenatal maternal characteristics**									
***Highest level of education***									
Bachelor’s degree or higher	1849 (40.1)	*Ref*			< 0.001	*Ref*			< 0.001
Diploma/Trade cert/NCEA 5–6	1390 (30.1)	0.105	0.032; 0.179	0.005		−0.110	−0.188; −0.033	0.005	
Secondary school/NCEA 1–4	1087 (23.6)	0.131	0.049; 0.212	0.002		−0.157	−0.243; −0.071	< 0.001	
No secondary school qualification	289 (6.3)	0.335	0.189; 0.481	< 0.001		−0.173	−0.328; −0.018	0.028	
***Prioritised ethnicity***									
European	2280 (49.3)	*Ref*			< 0.001	*Ref*			< 0.001
Māori	793 (17.1)	0.197	0.106; 0.288	< 0.001		−0.091	−0.187; 0.006	0.065	
Pacific	700 (15.1)	0.458	0.349; 0.567	< 0.001		−0.042	−0.157; 0.074	0.480	
Asian	686 (14.8)	0.292	0.192; 0.391	< 0.001		−0.465	−0.570; −0.359	< 0.001	
MELAA and others	165 (3.6)	0.169	0.016; 0.322	0.031		0.048	−0.114; 0.211	0.559	
***Age group***									
> 35 years	1274 (27.5)	*Ref*			0.001	*Ref*			0.663
20–34 years	3181 (68.7)	0.142	0.075; 0.208	< 0.001		0.036	−0.034; 0.107	0.315	
< 20 years	176 (3.8)	0.180	0.008; 0.353	0.041		0.018	−0.165; 0.201	0.846	
***BMI (kg/m***^***2***^***)***									
< 25.0	2396 (58.7)	*Ref*				*Ref*			0.003
25–29.99	951 (23.3)	−0.001	−0.073; 0.072	0.987	< 0.001	0.076	−0.001; 0.153	0.055	
> 30.0	737 (18.0)	0.101	0.017; 0.186	0.019		0.058	−0.032; 0.147	0.206	
***Physical activity before and during pregnancy***									
Moderate/vigorous physical activity before and during pregnancy	1387 (33.3)	*Ref*			0.042	*Ref*			0.001
Moderate/vigorous physical activity only before or during pregnancy	1101 (26.4)	−0.056	−0.130; 0.019	0.143		−0.011	0.090; 0.068	0.792	
No Moderate/vigorous physical activity before and during pregnancy	1675 (40.2)	−0.056	−0.124; 0.013	0.112		−0.086	−0.158; −0.013	0.021	
***“Junk” dietary pattern score***	5151 (100.0)	0.131	0.099; 0.164	< 0.001	–	−0.089	−0.124; −0.055	< 0.001	–
***“Health Conscious” dietary pattern score***	5151 (100.0)	−0.061	−0.093; −0.029	< 0.001	–	0.211	0.177; 0.245	< 0.001	–
***“Traditional/White” dietary pattern score***	5151 (100.0)	0.134	0.104; 0.173	< 0.001	–	0.022	−0.015; 0.058	0.243	–
***“Fusion” dietary pattern score***	5151 (100.0)	0.071	0.038; 0.104	< 0.001	–	0.027	−0.008; 0.062	0.125	–
**Maternal characteristics at the 24-month interview**									
**Neighbourhood deprivation (NZDep2006)**					0.012				
1–3 Least deprived	1647 (27.3)	*Ref*				*Ref*			0.386
4–7	2214 (36.7)	−0.028	−0.102; 0.045	0.450		−0.110	−0.188; −0.033	0.005	
8–10 Most deprived	2169 (36.0)	0.117	0.030; 0.203	0.008		−0.157	−0.243; −0.071	< 0.001	
**Currently smoking at least 1 cigarette/day**					< 0.001				
No	5343 (86.6)	*Ref*				*Ref*			0.158
Yes	824 (13.4)	0.343	0.243; 0.443	< 0.001		−0.101	−0.207; 0.004	0.060	

*Adjusted β* adjusted beta-coefficient, *Adjusted R*^*2*^ adjusted r-square of the model, *CI* confidence interval, *NCEA* National Certificate of Educational Achievement, *Ref* category of reference, *BMI* body mass index, *NZDep2006* neighbourhood deprivation index 2006.

^**#**^Model adjusted by child sex (female/male) and exact age at the 24-month interview in months (continuous).

Missing: Models at 24 months (n): Child age at the 24-month interview (^¶^); maternal education (1558); maternal ethnicity (1549); maternal age (1542); neighbourhood deprivation index (143); body mass index (2089).

maternal smoking patterns (^¶^); physical activity before/during pregnancy (2010); maternal dietary patterns-Junk, Health Conscious, Traditional/White and Fusion (1022).

*t-test, ** F-test.

^†^Average increase or decrease in the dietary patterns loading scores in relation to the categories of reference.

^¶^As per *Growing up in New Zealand* study anonymity requirement, “< 10” represents greater than zero and less than 10 children in the cell.

**Table 4:** Adjusted associations between the children's dietary patterns at the 54-month-interview and maternal sociodemographic and health behaviour characteristics (antenatally and at the 54-month-interview).

Covariates	N (%)	Refined high in sugar sodium and fat N = 5001; Adjusted R^2^ = 0.20^#^				Fruit and vegetables N = 5012; Adjusted R^2^ = 0.07^#^			
		Adjusted β^†^	95% CI	*P* value*	*P* value**	Adjusted β^†^	95% CI	*P* value*	*P* value**
**Antenatal maternal characteristics**									
***Highest level of education***									
Bachelor’s degree or higher	2441 (41.0)	*Ref*			< 0.001	*Ref*			0.840
Diploma/Trade cert/NCEA 5–6	1810 (30.4)	0.124	0.068; 0.181	< 0.001		0.001	−0.065; 0.067	0.971	
Secondary school/NCEA 1–4	1344 (22.6)	0.109	0.046; 0.173	< 0.001		0.021	−0.053; 0.096	0.571	
No secondary school qualification	354 (6.0)	0.217	0.102; 0.333	< 0.001		−0.010	−0.146; 0.125	0.880	
***Prioritised ethnicity***									
European	3062 (51.4)	*Ref*			< 0.001	*Ref*			0.803
Māori	1043 (17.5)	0.153	0.083; 0.222	< 0.001		−0.044	−0.125; 0.037	0.284	
Pacific	777 (13.0)	0.290	0.205; 0.375	< 0.001		−0.030	−0.129; 0.070	0.559	
Asian	867 (14.6)	0.131	0.054; 0.208	< 0.001		0.034	−0.056; 0.124	0.462	
MELAA and others	207 (3.5)	0.035	−0.085; 0.156	0.566		−0.004	−0.145; 0.137	0.958	
***Age group***									
> 35 years	1553 (26.0)	*Ref*			0.887	*Ref*			0.012
20–34 years	4171 (70.0)	0.027	−0.025; 0.080	0.307		0.075	0.014; 0.136	0.016	
< 20 years	239 (4.0)	0.028	−0.101; 0.158	0.668		0.175	0.028; 0.322	0.020	
***BMI (kg/m***^***2***^***)***									
< 25.0	3154 (59.4)	*Ref*			< 0.001	*Ref*			0.945
25–29.99	1210 (22.8)	0.024	−0.033; 0.081	0.412		0.038	−0.029; 0.104	0.265	
> 30.0	943 (17.8)	0.130	0.065; 0.195	< 0.001		−0.007	−0.083; 0.069	0.854	
***Physical activity before and during pregnancy***^†^									
Moderate/vigorous physical activity before and during pregnancy	1845 (34.1)	*Ref*			0.760	*Ref*			< 0.001
Moderate/vigorous physical activity only before or during pregnancy	1440 (26.6)	0.025	−0.033; 0.082	0.399		0.013	−0.054; 0.080	0.710	
No Moderate/vigorous physical activity before and during pregnancy	2128 (39.3)	0.015	−0.038; 0.068	0.580		−0.099	−0.160; −0.038	0.002	
***“Junk” dietary pattern score***	5013 (100.0)	0.212	0.186; 0.238	< 0.001	–	−0.040	−0.070; −0.009	0.010	–
***“Health Conscious” dietary pattern score***	5013 (100.0)	−0.057	−0.082; −0.032	< 0.001	–	0.166	0.137; 0.195	< 0.001	–
***“Traditional/White” dietary pattern score***	5013 (100.0)	0.141	0.114; 0.168	< 0.001	–	0.098	0.066; 0.130	< 0.001	–
***“Fusion” dietary pattern score***	5013 (100.0)	0.013	−0.006; 0.045	0.134	–	0.167	0.138; 0.197	< 0.001	–
**Maternal characteristics at the 54-month interview**									
***Neighbourhood deprivation (NZDep2013)***									
1–3 Least deprived	1744 (30.7)	*Ref*			0.237	*Ref*			0.090
4–7	2044 (36.0)	0.007	−0.047; 0.060	0.806		0.002	−0.060; 0.065	0.945	
8–10 Most deprived	1888 (33.3)	0.027	−0.036; 0.091	0.402		−0.048	−0.122; 0.027	0.207	
***Currently smoking at least 1 cigarette/day***									
No	5212 (86.9)	*Ref*		*Ref*	< 0.001	*Ref*			0.013
Yes	789 (13.1)	0.230	0.154; 0.307	< 0.001		0.082	−0.007; 0.172	0.072	

*Adjusted β* adjusted beta-coefficient, *Adjusted R*^*2*^ adjusted r-square of the model, *CI* confidence interval, *NCEA* National Certificate of Educational Achievement, *Ref* category of reference, *BMI* body mass index, *NZDep2013* neighbourhood deprivation index 2013.

^**#**^Model adjusted by child sex (female/male) and child exact age at the 54-month interview in months (continuous).

Missing-models at 54 months (n): Child’s sex (28); child age at the 54-month interview (0); maternal education (61); maternal ethnicity (54); maternal age (47); body mass index (703); neighbourhood deprivation index (334)

maternal smoking patterns (^¶^); physical activity before/during pregnancy (597); maternal dietary patterns-Junk, Health Conscious, Traditional/White and Fusion (997).

*t-test, ** F-test.

^†^Average increase or decrease in the dietary patterns loading scores in relation to the categories of reference.

^¶^As per *Growing up in New Zealand* study anonymity requirement, “< 10” represents greater than zero and less than 10 children in the cell.

In the adjusted models, children whose mothers had educational attainment lower than Bachelor's degree scored lower in *Fruit and vegetables* dietary pattern at 9- and 24-months. Children living in neighbourhoods belonging to deciles 8–10 of the NZDep (at 9-months) and deciles 4–10 (at 24-months) also scored lower in the *Fruit and vegetables* pattern. Children also scored lower in this pattern if their mothers: were from Māori or Pacific ethnicities (at 9-months) or Asian ethnicity (at 9- and 24-months); did not practice moderate/vigorous PA before and during pregnancy (at 24- and 54-months) and; had high antenatal scores in the “*Junk*” (at 9-,24- and 54-months) and the “*Traditional/White”* dietary patterns (at 9-months). At 54-months, children scored higher in the *Fruit and vegetables* pattern when their mothers were younger than 35 years of age. Higher children's scores in this dietary were also observed among those whose mothers had antenatal high scores in the “*Health conscious*” dietary pattern (all time-points), “*Fusion*” patterns (at 9- and 54-months) and “*Traditional/White*” pattern (at 54-months) (Tables 2, 3, 4).

## Discussion

This study provides the first NZ nationally generalizable information on the dietary patterns of under-five-year-olds. At 9-, 24-and 54-months two distinct dietary patterns were identified (*Refined high in sugar salt and fat* and *Fruit and vegetables).* At all time-points, the significant associations between the dietary patterns and maternal sociodemographic variables highlight the influence of inequities on children's dietary quality. Maternal health behaviours (diet quality in pregnancy and smoking habits) were also associated with children's poorer diet quality at all time-points.

The dietary patterns identified within *Growing up in New Zealand* cohort are similar to those described in the first years of life in other cohorts and surveys worldwide^9,11,13,19,23,27–29,34,52–56^. Most studies have identified at least two dietary patterns in this period of life, despite differences in naming and methods used to derive them. Overall, the patterns identified were those with: (1) high positive loading on fatty foods, refined grains, and highly processed and/or discretionary foods-with high contents of sodium, sugar and unhealthy fats and; (2) high loadings for fruits, vegetables and whole grains and low-fat protein products^9,13,19,27–29,34,52–56^. Studies involving infants and toddlers in the UK, Australia, Norway, and Japan identified two dietary patters, which were consistent with: core or recommended foods, and non-core or discretionary foods- high contents of sugar, sodium, and unhealthy fats^27–29,53,54^. The Southampton Women's Study identified a “*Guidelines*” pattern among 6- and 12-month-old children, which had high loadings in fruits, vegetables, and home prepared foods and “*Adult Foods*” pattern, with high consumption of chips, savoury snacks, and biscuits^27^. Secondary analysis of an Australian randomized trial and a longitudinal study found two patterns at ages 14- and 24-months. At 14- months, the “*Core foods*” pattern had high loadings in fruit, grains, vegetables, cheese, and nuts/seeds and at 24-months this pattern included fruit, vegetables, dairy, nuts/seeds, meat, and water. The “*Basic combination*” pattern had high loadings for white bread, milk, spreads, juice and ice-cream at 14-months and white bread, spreads, sweetened beverages, snacks, chocolate, and processed meat at 24-months^28^. Studies conducted with pre-schoolers and older children consistently identified a dietary pattern with high loadings in non-recommended foods-high amount of sugar, sodium, and healthy fats. The names commonly used to characterize the referred pattern included, but were not limited to “*Junk*”, “*Processed*” “*Processed and fast food*”, “High energy-dense foods”, “*Discretionary*”, “*Snacking*” and “*Sweet and fat*”^8,11,13,19,23,34,55,56^. Two NZ birth cohorts identified such a pattern among 3.5-, 6-, and 7-year-olds^13,55^. In the cohort nested within *Auckland Birthweight Collaborative* study, involving babies born small for gestational age (SGA) and appropriate for gestational age (AGA), the “*Junk*” dietary pattern identified at 3.5 and 7 years had high loadings for candy bars, hamburgers, soft drinks, and chips. Additional items with high loadings on foods where intakes were enquired only at 7 years were chocolate and lollies^55^. Among 1,142 six-year-olds who were part of the *Screening for Pregnancy Endpoints* (SCOPE) cohort- NZ, the '*Junk*' dietary pattern was characterized by high loadings in candy bars, potato crisps, lollies or sweets, sausage rolls, and bought cake or muffin^13^.

The associations found in this study indicate that there were ethnic and sociodemographic disparities in diet quality in early life in NZ and that children whose mothers were smokers or had lower diet quality also had low dietary quality during the first 5 years. We demonstrated that lower maternal education attainment was associated with higher scores in the *Refined high in sugar, salt, and fat* pattern at 9-, 24-and 54-months and lower scores in the *Fruit and vegetables* pattern at 9-and 24-months. These are not surprising findings and corroborate with those reported for 6-year-olds New Zealanders who scored higher in a “*Healthy*” pattern when their mothers had higher educational attainment and they scored higher in the “*Junk*” pattern when mothers had lower level of education^13^. Other cohorts have reported that children of mothers with lower education attainment scored higher in dietary patterns characterized by high intakes of foods with high amounts of energy, sugar and fat, and low amounts of fibre^18,22,53^ as well as lower in dietary patterns composed of fruit, vegetables, whole grains, and other relevant core foods^18,22,53^. Lower maternal education level was also associated with high trajectories of discretionary food intake in the first 3 years of life in the *Healthy Smiles Healthy Kids* (HSHK) birth-cohort study in South-Western Sydney, Australia^8^ The literature suggests that lower parental education may be related to lower literacy and knowledge about nutrition and healthier eating behaviours^23^.

Within *Growing Up in New Zealand* cohort, overall, despite of the variations on the magnitudes of associations across the three time-points, it was observed higher scores in the *Refined high in sugar, salt, and fat* pattern and lower in the *Fruit and vegetables* pattern among children from ethnicities other than European. These findings align with results from the NZ SCOPE cohort which identified that children of mothers Pacific or Indian ethnicities scored higher in the “*Junk*” dietary patterns at age 6 years while those from Indian and Māori mothers were less likely to score highly in the “*Healthy*” dietary pattern^13^. Maternal non-English speaking ethnicity was also associated with high trajectories of discretionary food intake among Australian children during the first 3 years of life^8^. Children whose mothers were younger at pregnancy scored higher in the *Refined high in sugar, salt, and fat* pattern at 9-and 24-months within *Growing Up in New Zealand* cohort, validating observations in NZ, Australia, and Brazil^8,20,22,28,53,55^. In this study, maternal smoking habits and diet quality in pregnancy were also associated with children's diet quality at all time-points. Significant associations between maternal smoking habits with infants' non-timely food introduction and lower diet quality at 9-,24-and 54- months^15–17^ have also been reported within the *Growing up in New Zealand c*ohort and within others two NZ cohorts^13,55^. Wall et al.^55^ and Flynn et al^13^ demonstrated that 6-year-olds New Zealanders were more likely to have higher scores in dietary patterns with high loading in foods high in energy, sugar, and fat when their mothers were smokers^13,55^. Previous observation suggests that smoking results from behavioural, psychosocial, and biological factors and that smoking mothers may be less likely to be health conscious and more likely to experience stress and lower social support^57^.

Another relevant finding of our study were the strong associations found between maternal diet quality in pregnancy and children diet quality at all time-points, where maternal high scores in the “*Junk*” pattern was positively associated with children's high scores in the *Refined high in sugar salt and fat* patterns and maternal high scores in the “*Health conscious*” pattern associated positively with children's scores in the *Fruit and vegetables* pattern. The strong influence of maternal dietary patterns on comparable dietary patterns for their children have been also reported in cohorts from the UK, Australia, and Portugal^7,19,23,27^ and authors have suggested that this may be due to the influence of maternal eating behaviours on children's food habits^19,23,27^^.^ Role modelling and the food environment at home is likely to be even more important in early life given higher dependency of children on their mothers^23^.

The identification of a dietary pattern loading high in refined foods and foods high in sugar, sodium and unhealthy fats within *Growing up in New Zealand* at the three time-points in early life is concerning as previous studies have showed significant associations between children's low diet quality in early life and adiposity later in childhood and adolescence^9,11,13,32,34,56^. This dietary pattern has also moderately stable trajectories through childhood and adolescence, with the intakes of the foods that load high on this pattern increasing with age^8,10,22,31,33,36,58^.

This study has limitations important to be mentioned. First, we do not have individual level deprivation measures. Data on deprivation are based on fined grained neighbourhood measures. It may explain why education presented a more consistent association with children’s dietary patterns. Second, the observational data used here make inference about causality difficult, because we have not looked at interplay of education, ethnicity, deprivation, and ‘health conscious’ maternal intake. It means that the ethnic differences observed in our study could be partially accounted for education, deprivation and vice-versa. At all time-points, the FFQs data were examined for potential implausible outliers of intake before running PCA. However, as the FFQs were not designed to capture the cohort's total energy intake, we could not with 100% certainty classify some outlier values of food servings as “implausible” and opted to keep all values of intake in the datasets. Additionally, the FFQs were administered to the child’s mother and represent an indirect measure of the child’s intake, which may be influenced by measurement error. Lastly, another limitation to consider is that PCA analysis uses the correlation matrix of food intake variables to identify common patterns of food consumption within the data to account for the largest amount of variation in diet within the studied population. Although widely employed to characterize children's dietary patterns, in PCA analyses the selection of the patterns is somewhat arbitrary and subjectivity relies on the author's interpretation^59^. As strengths of our study, we highlight the nationally generalizable characteristic of our cohort, representing the children from New Zealand. Comparisons between the *Growing up in New Zealand* cohort with contemporary national data for 2007–2010 indicated that there were only small differences between them (prevalence of low birth weight and preterm) which are unlikely to limit the external validity of findings to the wider NZ population. The large size of the cohort means that small variations in percentages (< 1%) between the cohort and national births reached statistical significance, but this statistical difference is unlikely to be of public health or clinical relevance^40^. The ability of *Growing up in New Zealand* cohort to track antenatal diet and diet in early life can provide insights into the potential long-term effects of early dietary choices on health outcomes in NZ. Our findings echo other previously published studies, adding further evidence for the need to intervene across several maternal determinants to improve infant and young child health outcomes through food choices and dietary patterns. Although the effects of maternal determinants on children’s dietary patterns appears to be quite robust, most studies describing this relationship have concentrated analysis on a single time-point, not assessing dietary intake patterns through the first years of life as presented in this work.

## Conclusion

Findings from this study indicate that diet quality in early life may be improved if sociodemographic disparities are narrowed in NZ. This may include the facilitation of maternal access to education and promoting culturally safe and integrated structural approaches to achieve improvements in early life diet quality. Examples of structural approaches include government led interventions for better affordability of healthy diets and to limit the availability and the marketing of junk foods (high content of added sugar, sodium and unhealthy fats) to children. NZ has currently no government-led mandatory actions or legislation to improve the healthiness of the food environments and to limit children's exposure to unhealthy foods^60^. Maternal smoking cessation and promotion of healthy diets among women of reproductive may also elicit long-term impacts on children’s diet quality, and health and wellbeing.

### Supplementary Information

Supplementary Information.

## Data Availability

The anonymized data that support the findings of this study are available from the Growing up in New Zealand Cohort Study, but restrictions apply to the availability of these data, which were used under license for the current study, and so are not publicly available. Data are however available from the corresponding author (Dr Teresa Gontijo de Castro) upon reasonable request and with permission of the Growing Up in New Zealand Data Access Committee (email: dataaccess@growingup.co.nz).
