# Supplementary material for: Maternal determinants of dietary patterns in infancy and early childhood in the Growing up in New Zealand cohort
Source: Sci Rep. 2023 Dec 20;13:22754. doi: 10.1038/s41598-023-49986-2 (PMC10733397; doi:10.1038/s41598-023-49986-2)
Supplement: Supplementary file 1 — Supplementary Information. [file 41598_2023_49986_MOESM1_ESM.docx]

**Table S1.** List of food items and groups aggregated according to the National Food and Nutrition Guidelines * that were in place at the 9-, 24- and 54-month interviews.

| **9-month-interview**  **Daily frequency of intake** | **24-month-interview**  **Daily servings** | **54-month-interview**  **Daily servings** |
| --- | --- | --- |
| **Vegetables** (raw or cooked) | **Green leafy vegetables** (lettuce, cabbage, bok choy, spinach, brussel sprouts, taro leaves, pele leaves, puha or any other green leafy vegetable). | **Green leafy vegetables** (lettuce, cabbage, bok choy, spinach, brussel sprouts, taro leaves, pele leaves, puha or any other green leafy vegetable). |
|  | **Other types of vegetables** (Peas, green beans, mushrooms; Potatoes, kumara, pumpkin, yams, taro, sweet potatoes; Carrots; Broccoli, cauliflower; Sweetcorn; Peppers/tomatoes).  ***Observation:*** *It excludes hot hips, French fries, wedges, or kumara chips.* | **Other types of vegetables** (Peas, green beans, mushrooms; Potatoes, kumara, pumpkin, yams, taro, sweet potatoes; Carrots; Broccoli, cauliflower; Sweetcorn; Peppers/tomatoes; Avocados).  ***Observation:*** *It excludes hot hips, French fries, wedges, or kumara chips.* |
| **Fruit** (Fresh or canned) | **Citrus fruits, such as oranges, lemons, grapefruit** | **Citrus fruits, such as oranges, lemons, grapefruit** |
|  | **Other types of fruit** (Apples, pears; Bananas; Peaches, nectarines, melon, lychees, paw-paw; Strawberries, raspberries, blueberries, mango, kiwi fruit; Plums, cherries, grapes).  ***Observation:*** *It excludes dried fruits and fruit juices. The Guidelines* that were in place when the 24- and 54-month interviews took place accounted as “fruit” the fruit juices and dried juices (with a cap of 1 serve/day). However, because of the content of sugars of these items, they were not included under “Fruits” in this study. Fruit juices were included under “Inappropriate drinks” and dried fruits were not included in the principal component analyses.* | **Other types of fruit** (Apples, pears; Bananas; Peaches, nectarines, melon, lychees, paw-paw; Strawberries, raspberries, blueberries, mango, kiwi fruit; Plums, cherries, grapes).  ***Observation:*** *It excludes dried fruits and fruit juices. The Guidelines* that were in place when the 24- and 54-month interviews took place accounted as “fruit” the fruit juices and dried juices (with a cap of 1 serve/day). However, because of the content of sugars of these items, they were not included under “Fruits” in this study. Fruit juices were included under “Inappropriate drinks” and dried fruits were not included in the principal component analyses.* |
| **Breads and cereals** (Bread or toast; Other cereal) | **Breads and cereals-white, refined options** (White bread; Roti, naan pitta; Other bread; Noodles or rice or pasta, rice porridge; Semolina, tapioca, sago; Other cereals, such as cornflakes, rice, puffed wheat, nutrigrain; Cakes or biscuits). | **Breads and cereals-white, refined options** (White bread; Roti, naan pitta; Other bread; Noodles or rice or pasta, rice porridge; Semolina, tapioca, sago; Other cereals, such as cornflakes, rice, puffed wheat, nutrigrain; Cakes or biscuits; Crackers). |
| **Baby foods-iron enriched** (Baby breakfast cereal; Rusks; Baby rice) | **Breads and cereals-brown, wholegrain options** (High fibre white bread; Brown bread, wholemeal or wholegrain bread; High fibre cereals, such as muesli, porridge, weetbix, branflakes) | **Breads and cereals-brown, wholegrain options** (High fibre white bread; Brown bread, wholemeal or wholegrain bread; High fibre cereals, such as muesli, porridge, weetbix, branflakes |
| **Milk products** (Milk puddings, rice pudding, yoghurt, custards) | **Milk, milk products or suitable alternatives- whole milk, non-reduced fat options** (Whole or standard milk -dark blue or silver; Cheese -including paneer, cottage cheese; Yoghurt) | **Milk, milk products or suitable alternatives- whole milk, non-reduced fat options** (Whole or standard milk -dark blue or silver; Cheese -including paneer, cottage cheese; Yoghurt) |
|  | **Milk and milk products- skim/trim milk, low-fat options** (Reduced fat-light blue; Skim or trim-green or yellow; Soymilk; Other milk -such as rice, goat`s milk) | **Milk and milk products- skim/trim milk, low-fat options** (Reduced fat-light blue; Skim or trim-green or yellow; Soymilk; Other milk -such as rice, goat`s milk) |
| **Milks: Infant suitable formula milks**  (cow`s, goat`s, soy`s, follow-on, hypo-allergenic and other infant formula milk)  ***Observation:*** *The Guidelines for infants recommend babies to be breastfed or to be given suitable infant formula milk if breastfeeding cannot be implemented.* | **Milk: Toddler formula milk**  ***Observation:*** *The Guidelines for toddlers discourage the use of toddler formula milks.* | --- |
| **Protein group: Meat, chicken or meat dishes; Fish or fish dishes** (fresh and canned); **Shellfish; Eggs; Soyfoods, tofu, soy desserts; Nuts or peanut butter.** | **Protein group: Lean meat, poultry, fish, shellfish, eggs, legumes, nuts and seeds (**Red meat or dishes containing red meat such as beef, pork, mutton, lamb and goat; Chicken or dishes containing chicken; Seafood such as fish or shellfish, fresh or frozen; Alternative protein such as legumes, nuts (used in a meal), tofu, textured vegetable protein; Eggs).  ***Observation:*** *As the intake of “Peanut butter/Nutella” was low within the GUiNZ cohort at the 24-and 54-month interviews^16^, this item was treated as “spreads” and not included in the principal component analyses because the Guidelines for pre-schoolers* did not make recommendation for spreads.* | **Protein group: Lean meat, poultry, fish, shellfish, eggs, legumes, nuts and seeds (**Red meat or dishes containing red meat such as beef, pork, mutton, lamb and goat; Chicken or dishes containing chicken; Seafood such as fish or shellfish, fresh or frozen; Alternative protein such as legumes, nuts (used in a meal), tofu, textured vegetable protein; Eggs).  ***Observation:*** *As the intake of “Peanut butter/Nutella” was low within the GUiNZ cohort at the 24-and 54-month interviews^16^ , this item was treated as “spreads” and not included in the principal* *component analyses because the Guidelines for pre-schoolers* did not make recommendation for spreads.* |
|  | **Processed meats** (such as ham, bacon, pastrami, salami, sausages, chicken nuggets, luncheon, canned corned beef; Processed fish as tinned fish or fish sachets) | **Processed meats** (such as ham, bacon, pastrami, salami, sausages, chicken nuggets, luncheon, canned corned beef; Processed fish as tinned fish or fish sachets) |
| **Inappropriate drinks -not recommended to infants** (soft drinks, fruit juices-includes watered down juices, coffee, tea, herbal drinks | **Inappropriate drinks- high content of added sugars** (Soft drinks or energy drinks; Soft drinks that don’t contain sugar – this includes diet varieties; Fruit juice and drinks) | **Inappropriate drinks- high content of added sugars** (Soft drinks or energy drinks; Soft drinks that don’t contain sugar – this includes diet varieties; Fruit juice and drinks) |
| **Inappropriate foods- high content of added sugars** (sweets, chocolates and sweets) | **Inappropriate foods- high content of added sugars** (Confectionary, lollies, sweets and chocolate; Ice-cream).  ***Observation:*** *The Guidelines* that were in place accounted “Ice-cream” under “Milk and Milk products. However, because of the content of sugars of this item, ice-cream was included under “Inappropriate foods” in this study.* | **Inappropriate foods- high content of added sugars** (Confectionary, lollies, sweets and chocolate; Ice-cream).  ***Observation:*** *The Guidelines* that were in place accounted “Ice-cream” under “Milk and Milk products. However, because of the content of sugars of this item, ice-cream was included under “Inappropriate foods” in this study.* |
| **Inappropriate foods- high content of sodium and fat** (hot chips, potato crisps) | **Inappropriate foods- high content of sodium and fat, especially saturated fat** (Hot chips, French fries, wedges, or kumara chips; Battered or fried fish or seafood; Takeaways from places like McDonalds, KFC, Burger King, Pizza shops or fast -food outlets; Snacks such as crisps, corn chips & similar chips, muesli bars, popcorn, or nuts as a snack). | **Inappropriate foods- high content of sodium and fat, especially saturated fat** (Hot chips, French fries, wedges, or kumara chips; Battered or fried fish or seafood; Takeaways from places like McDonalds, KFC, Burger King, Pizza shops or fast -food outlets; Snacks such as crisps, corn chips & similar chips, muesli bars, popcorn, or nuts as a snack). |

* New Zealand National Food and Nutrition Guidelines for Infants^42^ and the New Zealand National Food and Nutrition Guidelines for Pre-schoolers^43.^

**Table S2.** Characteristics of the children and their mothers included in the analyses of dietary patterns at 9 (n=6,259), 24 (n=6,292) and 54 months (n= 6,131).

| **Children`s and mothers` characteristics** | **9-month interview** | **24-month interview** | **54-month interview** |
| --- | --- | --- | --- |
|  | **Children (perinatal, at 24-and 54-month interviews)** | | |
| **Exact age at the interview-months: mean (SD)** | 9.0 (0.9) | 24.3 (2.0) | 54.0 (1.6) |
| **Gestational age-weeks: mean (SD)** | 39.1 (1.9) | -- | -- |
| **Birth weight-grams: mean (SD)** | 3490 (583.6) | -- | -- |
| **Singletons/twins-triplets: n(%)**  Singletons  Twins/triplets | 6103 (97.5)  156 (2.5) | 6139 (97.5)  153 (2.5) | 5984 (97.6)  147 (2.4) |
| **Sex: n(%)**  Female  Male | 3018 (48.4)  3218 (51.6) | 3032 (48.4)  3233(51.6) | 2961 (48.5)  3141 (51.5) |
| **Breastfeeding duration: n(%)**  Never  <6 months  >6 months | 195 (3.1)  1955 (31.3)  4089 (65.5) | -- | -- |
|  | **Mothers (antenatal interview)** | | |
| ***Highest level of education:* n(%)**  Bachelor’s degree or higher  Diploma/Trade cert/NCEA 5-6  Secondary school/NCEA 1-4  No secondary school qualification | 2435 (39.6)  1893 (30.8)  1425 (23.1)  400 (6.5) | 1859 (40.0)  1399 (30.1)  1095 (23.6)  292 (6.3) | 2460 (41.1)  1820 (30.3)  1353 (22.6)  358 (6.0) |
| ***Age group:* n(%)**  >35 years  20-34 years  <20 years | 1586 (25.7)  4305 (69.80)  278 (4.5) | 1286 (27.6)  3198 (68.6)  177 (3.8) | 1570 (26.1)  4196 (69.9)  239 (4.0) |
| ***Prioritised ethnicity:* n(%)**  European  Māori  Pacific  Asian  MELAA and others | 3075 (49.9)  1106 (17.9)  850 (13.8)  923 (15.0)  209 (3.4) | 2300 (49.4)  798 (12.6)  700 (15.0)  690 (14.8)  166 (3.6) | 3087 (51.5)  1051 (17.5)  781 (13.0)  869 (14.5)  210 (3.5) |
| ***Neighbourhood deprivation (NZDep2006)***: **n(%)**  1-3 Least deprived  4-7  8-10 Most deprived | 1580 (25.6)  2304 (37.4)  2283 (37.0) | -- | -- |
| ***BMI (kg/m^2^):* n(%)**  <25.0  25-29.99  >30.0 | 3230 (59.1)  1232 (22.6)  1000 (18.3) | 2407 (58.6)  956 (23.2)  747 (18.2) | 3173 (59.4)  1220 (22.8)  951 (17.8) |
| ***Physical activity before and during pregnancy* n(%)**  Moderate/vigorous physical activity before and during pregnancy  Moderate/vigorous physical activity only before or during pregnancy  No moderate/vigorous physical activity before and during pregnancy | 1878 (33.7)  1483 (26.6)  2214 (39.7) | 1393 (33.3)  1107 (26.4)  1689 (40.3) | 1854 (34.0)  1449 (26.6)  2150 (39.4) |
| ***Smoking habits before and during pregnancy:* n(%)**  Non-smokers before and during pregnancy  Stopped smoking during pregnancy  Smokers before and during pregnancy | 4483 (80.7)  541 (9.7)  534 (9.6) | -- | -- |
| ***Dietary patterns (loading scores):* n(%)** | 5134 (100.0) | 5184 (100.0) | 5048 (100) |
|  | **Mothers (24-month interview)** | | |
| **Neighbourhood deprivation (NZDep2006): n(%)**  1-3 Least deprived  4-7  8-10 Most deprived | -- | 1661 (27.4)  2231 (36.8)  2177 (35.9) | -- |
| **Currently smoking at least 1 cigarette/day: n(%)**  No  Yes | -- | 5375 (86.6)  831 (13.4) | -- |
|  | **Mothers (54-month interview)** | | |
| **Neighbourhood deprivation (NZDep20013): n(%)**  1-3 Least deprived  4-7  8-10 Most deprived | -- | -- | 1753 (30.7)  2061 (36.1)  1901 (33.2) |
| **Currently smoking at least 1 cigarette/day: n(%)**  No  Yes | -- | -- | 5249 (86.9)  794 (13.1) |

NCEA: National Certificate of Educational Achievement, NZDep2006: neighbourhood deprivation index 2006, BMI: body mass index, NZDep2013: neighbourhood deprivation index 2013.Missing at the 9-month interview: Child’s sex (23); fetal count (0); child age at the 9-month interview (^¶^); gestational age (29); birth weight (24); breastfeeding duration (20); maternal education (106); maternal ethnicity (96); maternal age; (90); neighbourhood deprivation index (92); maternal smoking patterns (701); maternal dietary patterns (1125). Missing at the 24-month interview: Child’s sex (27); fetal count (0); child age at the 24-month interview (^¶^); maternal education (1647); maternal ethnicity (1638); maternal age; (45); neighbourhood deprivation index (223); maternal smoking patterns (86); maternal dietary patterns (1108).Missing at the 54-month interview: Child’s sex (29); fetal count (0); child age at the 54-month interview (0); maternal education (140); maternal ethnicity (133); maternal age; (126); neighbourhood deprivation index (415); maternal smoking patterns (88); maternal dietary patterns (1083). ^¶^As per *Growing up in New Zealand* study anonymity requirement, “<10” represents greater than zero and less than 10 children in the cell.

| 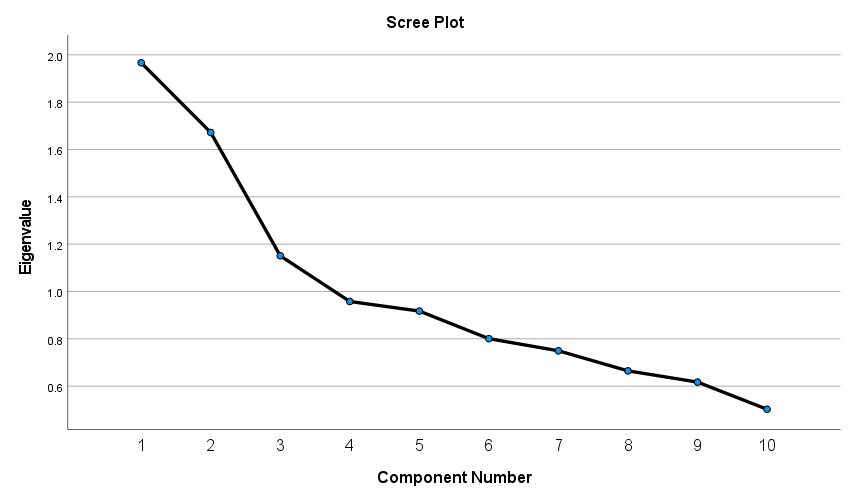 | 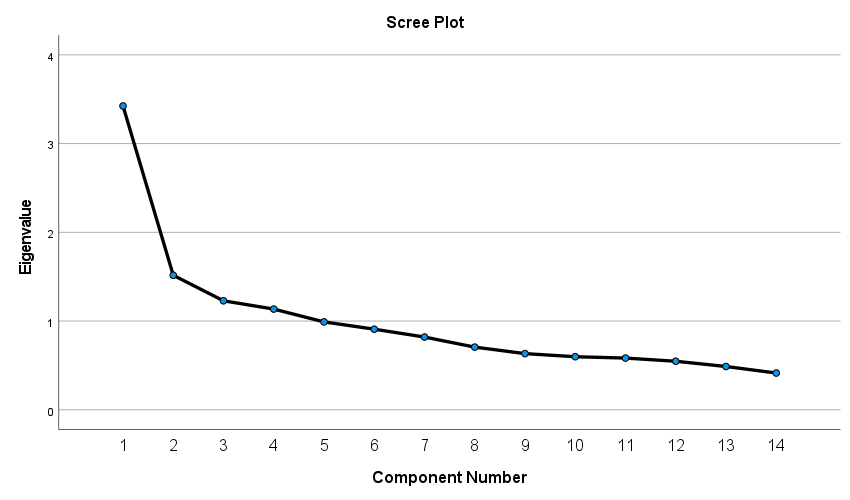  **24-month interview**  **54-month interview**  **9-month interview** | 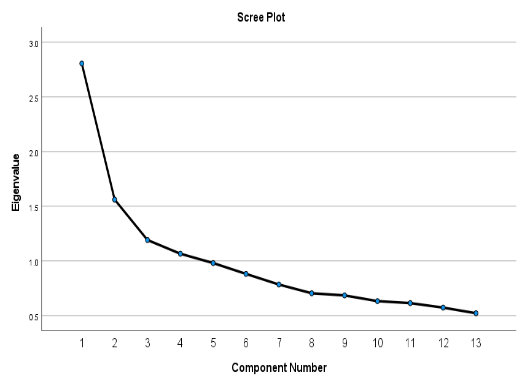 |
| --- | --- | --- |

**Figure S1.** Scree plots for the dietary patterns identified at the 9-, 24- and 54-month-interviews.

**Table S3.** Unadjusted associations between the infants` dietary patterns at the 9-month-interview and their perinatal and maternal sociodemographic and health behaviour characteristics (all cohort, N=6,167).

| **Covariates** | **N (%)**^a^ | **Refined high in sugar sodium and fat** | | | | **Fruit and vegetables** | | | |
| --- | --- | --- | --- | --- | --- | --- | --- | --- | --- |
|  |  | **Unadjusted β**^†^ | **95%CI** | **P value*** | **P value**** | **Unadjusted**  **β**^†^ | **95%CI** | **P value*** | **P value**** |
|  |  | **Child characteristics** | | | | | | | |
| **Sex**  Girl  Boy | 2970 (48.3)  3179 (51.7) | *Ref.*  0.033 | -0.017; 0.082 | 0.199 | 0.199 | *Ref.*  0.067 | 0.017; 0.117 | 0.009 | 0.009 |
| ***Birth weight (grams)*** | 6148 (100.0) | 0.000 | 0.000; 0.000 | 0.408 | -- | 0.000 | 0.000; 0.000 | <0.001 | -- |
| ***Gestational age (weeks)*** | 6143 (100) | 0.016 | 0.002; 0.030 | 0.025 | -- | 0.018 | 0.003; 0.032 | 0.015 | -- |
| ***Age at the 9-month interview (months)*** | 6163 (100.0) | 0.324 | 0.296; 0.352 | <0.001 | -- | 0.058 | 0.029; 0.087 | <0.001 | -- |
| ***Breastfeeding duration***  >6 months  < 6 months  Never breastfed | 4055 (65.9)  1910 (31.0)  188 (3.1) | *Ref.*  0.233  0.300 | 0.179; 0.287  0.155; 0.446 | <0.001  <0.001 | <0.001 | *Ref.*  -0.389  -0.491 | -0.443; -0.336  -0.635; -0.347 | <0.001  <0.001 | <0.001 |
|  |  | **Antenatal maternal characteristics** | | | | | | | |
| ***Highest level of education***  Bachelor’s degree or higher  Diploma/Trade cert/NCEA 5-6  Secondary school/NCEA 1-4  No secondary school qualification | 2413 (39.5)  1881 (30.8)  1416 (23.2)  396 (6.5) | *Ref.*  0.319  0.348  0.940 | 0.260; 0.377  0.285; 0.412  0.836; 1.04 | <0.001  <0.001  <0.001 | <0.001 | *Ref.*  -0.442  -0.529  -0.758 | -0.501; -0.384  -0.592; -0.466  -0.861; -0.655 | <0.001  <0.001  <0.001 | <0.001 |
| ***Prioritised ethnicity***  European  Māori  Pacific  Asian  MELAA and others | 3041 (49.7)  1101 (18.0)  849 (13.9)  919 (15.0)  206 (3.4) | *Ref.*  0.612  0.563  0.043  0.080 | 0.546; 0.679  0.489; 0.636  -0.028; 0.155  -0.057; 0.216 | <0.001  <0.001  0.232  0.253 | <0.001 | *Ref.*  -0.545  -0.507  -0.354  -0.020 | -0.612; -0.478  -0.581; -0.433  -0.426; -0.282  -0.158; 0.177 | <0.001  <0.001  <0.001  0.117 | <0.001 |
| ***Age group***  >35 years  20-34 years  <20 years | 1568 (25.6)  4277 (69.9)  277 (4.5) | *Ref.*  0.248  1.027 | 0.191; 0.305  0.902; 1.152 | <0.001  <0.001 | <0.001 | *Ref.*  -0.197  -0.661 | -0.255; -0.140  -0.788; -0.534 | <0.001  <0.001 | <0.001 |
| ***Neighbourhood deprivation (NZDep2006)***  1-3 Least deprived  4-7  8-10 Most deprived | 1567 925.6)  2281 (37.3)  2272 (37.1) | *Ref.*  0.078  0.463 | 0.015; 0.141  0.400; 0.527 | 0.016  <0.001 | <0.001 | *Ref.*  -0.118  -0.455 | -0.182; -0.055  -0.518; -0.391 | <0.001  <0.001 | <0.001 |
| ***BMI (kg/m^2^)***  <25.0  25-29.99  >30.0 | 3208 (59.2)  1220 (22.5)  993 (18.3) | *Ref.*  -0.001  0.136 | -0.066; 0.063  0.066; 0.206 | 0.967  <0.001 | <0.001 | *Ref.*  -0.071  -0.167 | -0.135; -0.007  -0.237; -0.097 | 0.031  <0.001 | <0.001 |
| ***Smoking before and during pregnancy***  Non-smokers before and during pregnancy  Stopped smoking during pregnancy  Smokers before and during pregnancy | 4444 (80.6)  537 (9.7)  532 (9.6) | *Ref.*  0.249  0.762 | 0.162; 0.336  0.675;0.849 | <0.001  <0.001 | <0.001 | *Ref.*  -0.336  0.511 | -0.424; -0.248  -0.599; -0.422 | <0.001  <0.001 | <0.001 |
| ***Physical activity before and during pregnancy***^†^  Moderate/vigorous physical activity before and during pregnancy  Moderate/vigorous physical activity only before or during pregnancy  No Moderate/vigorous physical activity before and during pregnancy | 1868 (33.8)  1469 (26.6)  2193 (39.6) | *Ref.*  -0.152  -0.093 | -0.217; -0.088  -0.151; -0.036 | <0.001  0.002 | 0.252 | *Ref.*  0.029  -0.079 | -0.035; 0.094  -0.137; -0.021 | 0.374  0.007 | 0.013 |
| ***“Junk” dietary pattern score*** | 5115 (100.0) | 0.208 | 0.182; 0.234 | <0.001 | -- | -0.089 | -0.117; -0.061 | <0.001 | --- |
| ***“Health Conscious” dietary pattern score*** | 5115 (100.0) | -0.093 | -0.119; -0.066 | <0.001 | -- | 0.235 | 0.208; 0.261 | <0.001 | --- |
| ***“Traditional/White” dietary pattern score*** | 5115 (100.0) | 0.233 | 0.207; 0.259 | <0.001 | -- | -0.190 | -0.217; -0.162 | <0.001 | --- |
| ***“Fusion” dietary pattern score*** | 5115 (100.0) | 0.015 | -0.012; 0.042 | 0.268 | -- | 0.047 | 0.019; 0.075 | <0.001 | --- |

Unadjusted.β, unadjusted beta-coefficient; CI: confidence interval, NCEA, National Certificate of Educational Achievement, NZDep2006, Ref: category of reference, neighbourhood deprivation index 2006, BMI: body mass index.

Missing: models at 9 months (n): Child’s sex (18); child age at the 9-month interview (^¶^); gestational age (24); breastfeeding duration (14); maternal education (61); maternal ethnicity (51); maternal age; (45); neighbourhood deprivation index (47); maternal smoking patterns (654); maternal dietary patterns Junk, Health Conscious, Traditional/White and Fusion (1052).

*t-test,** F-test

† Average increase or decrease in the dietary patterns loading scores in relation to the categories of reference.

^¶^As per *Growing up in New Zealand* study anonymity requirement, “<10” represents greater than zero and less than 10 children in the cell.

**Table S4.** Unadjusted associations between the children`s dietary patterns at the 24-month-interview and the maternal sociodemographic and health behaviour characteristics (all cohort, N=6,173).

| **Covariates** | **N (%)**^a^ | **Refined high in sugar sodium and fat** | | | | **Fruit and vegetables** | | | |
| --- | --- | --- | --- | --- | --- | --- | --- | --- | --- |
|  |  | **Unadjusted. β**^†^ | **95%CI** | **P value*** | **P value**** | **Unadjusted. β**^†^ | **95%CI** | **P value*** | **P value**** |
|  |  | **Child characteristics** | | | | | | | |
| **Sex**  Girl  Boy | 2961 (48.2)  3186 (51.8) | *Ref.*  0.022 | -0.028; 0.073 | 0.388 | 0.388 | *Ref.*  0.024 | -0.027; 0.074 | 0.355 | 0.355 |
| ***Age at the 24-month interview (months)*** | 6158 (100.) | 0.076 | 0.064; 0.089 | <0.001 | -- | -0.010 | -0.022; 0.003 | 0.135 | -- |
|  |  | **Antenatal maternal characteristics** | | | | | | | |
| ***Highest level of education***  Bachelor’s degree or higher  Diploma/Trade cert/NCEA 5-6  Secondary school/NCEA 1-4  No secondary school qualification | 1849 (40.1)  1390 (30.1)  1087 (23.6)  289 (6.3) | *Ref.*  0.412  0.558  0.960 | 0.341; 0.483  0.481; 0.634  0.833; 1.09 | <0.001  <0.001  <0.001 | <0.001 | *Ref.*  -0.158  -0.245  -0.310 | -0.228; -0.087  -0.321; -0.169  -0.435; -0.184 | <0.001  <0.001  <0.001 | <0.001 |
| ***Prioritised ethnicity***  European  Māori  Pacific  Asian  MELAA and others | 2280 (49.3)  793 (17.1)  700 (15.1)  686 (14.8)  165 (3.6) | *Ref.*  0.637  1.079  0.427  0.199 | 0.557; 0.717  0.996; 1.163  0.343; 0.511  0.044; 0.355 | <0.001  <0.001  <0.001  0.012 | <0.001 | *Ref.*  -0.241  -0.212  -0.624  -0.035 | -0.321; -0.160  -0.296; -0.127  -0.709; -0.539  -0.192; 0.123 | <0.001  <0.001  <0.001  0.665 | <0.001 |
| ***Age group***  >35 years  20-34 years  <20 years | 1274 (27.5)  3181 (68.7)  176 (3.8) | *Ref.*  0.337  1.02 | 0.269; 0.404  0.856; 1.18 | <0.001  <0.001 | <0.001 | *Ref.*  -0.088  -0.347 | -0.154; -0.022  -0.508; -0.187 | 0.009  <0.001 | <0.001 |
| ***BMI (kg/m^2^)***  <25.0  25-29.99  >30.0 | 2396 (58.7)  951 (23.3)  737 (18.0) | *Ref.*  -0.009  0.265 | -0.086; 0.068  0.180; 0.351 | 0.818  <0.001 | <0.001 | *Ref.*  0.065  0.074 | -0.009; 0.140  -0.008; 0.156 | 0.086  0.078 | 0.297 |
| ***Physical activity before and during pregnancy***^†^  Moderate/vigorous physical activity before and during pregnancy  Moderate/vigorous physical activity only before or during pregnancy  No Moderate/vigorous physical activity before and during pregnancy | 1387 (33.3)  1101 (26.4)  1675 (40.2) | *Ref.*  -0.121  -0.027 | -0.201; -0.042  -0.097; 0.043 | 0.003  0.457 | 0.903 | *Ref.*  -0.021  -0.180 | -0.097; 0.055  -0.247; -0.113 | 0.588  <0.001 | <0.001 |
| ***“Junk” dietary pattern score*** | 5151 (100.0) | 0.193 | 0.166; 0.220 | <0.001 | -- | -0.067 | -0.096; -0.039 | <0.001 | --- |
| ***“Health Conscious” dietary pattern score*** | 5151 (100.0) | 0.141 | -0.167; -0.114 | <0.001 | -- | 0.260 | 0.234; 0.286 | <0.001 | --- |
| ***“Traditional/White” dietary pattern score*** | 5151 (100.0) | 0.274 | 0.248; 0.300 | <0.001 | -- | -0.011 | -0.038; 0.017 | 0.435 | --- |
| ***“Fusion” dietary pattern score*** | 5151 (100.0) | 0.105 | 0.077; 0.132 | <0.001 | -- | -0.007 | -0.035; 0.021 | 0.621 | --- |
|  |  | **Maternal characteristics at the 24-month interview** | | | | | | | |
| ***Neighbourhood deprivation (NZDep2006)***  1-3 Least deprived  4-7  8-10 Most deprived | 1647 (27.3)  2214 (36.7)  2169 (36.0) | *Ref.*  0.095  0.599 | 0.034; 0.155  0.538; 0.660 | 0.002  <0.001 | <0.001 | *Ref.*  -0.065  -0.151 | -0.128; -0.002  -0.214; -0.088 | 0.042  <0.001 | <0.001 |
| ***Currently smoking at least 1 cigarette/day***  No  Yes | 5343 (86.6)  824 (13.4) | *Ref.*  0.692 | 0.620; 0.764 | <0.001 | <0.001 | *Ref.*  -0.198 | -0.271; -0.124 | <0.001 | <0.001 |

Unadjusted.β, unadjusted beta-coefficient; CI: confidence interval, NCEA, National Certificate of Educational Achievement, NZDep2006, Ref: category of reference, neighbourhood deprivation index 2006, BMI: body mass index.

Missing: Models at 24 months (n): Child’s sex (26); child age at the 24-month interview (^¶^); maternal education (1558); maternal ethnicity (1549); maternal age (1542);

neighbourhood deprivation index (143); body mass index (2089); maternal smoking patterns (^¶^); physical activity before/during pregnancy (2010); maternal dietary patterns Junk, Health Conscious, Traditional/White and Fusion (1022).

*t-test,** F-test

† Average increase or decrease in the dietary patterns loading scores in relation to the categories of reference.

^¶^As per *Growing up in New Zealand* study anonymity requirement, “<10” represents greater than zero and less than 10 children in the cell.

**Table S5.** Unadjusted associations between the children`s dietary patterns at the 54-month-interview and the maternal sociodemographic and health behaviour characteristics (all cohort, N=6,010).

| **Covariates** | **N (%)**^a^ | **Refined high in sugar sodium and fat** | | | | **Fruit and vegetables** | | | |
| --- | --- | --- | --- | --- | --- | --- | --- | --- | --- |
|  |  | **Unadjusted. β**^†^ | **95%CI** | **P value*** | **P value**** | **Unadjusted. β**^†^ | **95%CI** | **P value*** | **P value**** |
|  |  |  | | | | **Child characteristics** | | |  |
| **Sex**  Girl  Boy | 2890 (48.3)  3092 (51.7) | *Ref.*  0.151 | 0.103; 0.199 | <0.001 | <0.001 | *Ref.*  0.034 | -0.017; 0.085 | 0.192 | 0.192 |
| ***Age at the 54-month interview (months)*** | 6010 (100.0) | 0.079 | 0.063; 0.094 | <0.001 | -- | 0.007 | -0.010; 0.023 | 0.416 | --- |
|  |  | **Antenatal maternal characteristics** | | | | | | | |
| ***Highest level of education***  Bachelor’s degree or higher  Diploma/Trade cert/NCEA 5-6  Secondary school/NCEA 1-4  No secondary school qualification | 2441 (41.0)  1810 (30.4)  1344 (22.6)  354 (6.0) | *Ref.*  0.374  0.424  0.822 | 0.319; 0.430  0.363; 0.485  0.720; 0.925 | <0.001  <0.001  <0.001 | <0.001 | *Ref.*  -0.005  -0.002  0.034 | -0.065; 0.056  -0.068; 0.064  -0.078; 0.145 | 0.878  0.953  0.553 | 0.767 |
| ***Prioritised ethnicity***  European  Māori  Pacific  Asian  MELAA and others | 3062 (51.4)  1043 (17.5)  777 (13.0)  867 (14.6)  207 (3.5) | *Ref.*  0.536  0.762  0.138  0.011 | 0.473; 0.600  0.690; 0.833  0.070; 0.206  -0.116; 0.138 | <0.001  <0.001  <0.001  0.865 | <0.001 | *Ref.*  0.021  0.082  0.060  0.065 | -0.049; 0.091  0.004; 0.161  -0.015; 0.136  -0.076; 0.206 | 0.555  0.040  0.116  0.365 | 0.030 |
| ***Age group***  >35 years  20-34 years  <20 years | 1553 (26.0)  4171 (70.0)  239 (4.0) | *Ref.*  0.179  0.732 | 0.124; 0.233  0.605; 0.860 | <0.001  <0.001 | <0.001 | *Ref.*  0.058  0.077 | 0.000; 0.117  -0.059; 0.213 | 0.049  0.267 | 0.049 |
| ***BMI (kg/m^2^)***  <25.0  25-29.99  >30.0 | 3154 (59.4)  1210 (22.8)  943 (17.8) | *Ref.*  0.022  0.269 | -0.039; 0.083  0.202; 0.336 | 0.486  <0.001 | <0.001 | *Ref.*  -0.047  -0.064 | -0.112; 0.017  -0.136; 0.007 | 0.151  0.076 | 0.050 |
| ***Physical activity before and during pregnancy***^†^  Moderate/vigorous physical activity before and during pregnancy  Moderate/vigorous physical activity only before or during pregnancy  No Moderate/vigorous physical activity before and during pregnancy | 1845 (34.1)  1440 (26.6)  2128 (39.3) | *Ref.*  -0.054  -0.001 | -0.116; 0.007  -0.056; 0.055 | 0.085  0.978 | 0.247 | *Ref.*  -0.020  -0.146 | -0.086; 0.045  -0.204; -0.088 | 0.538  <0.001 | <0.001 |
| ***“Junk” dietary pattern score*** | 5013 (100.0) | 0.262 | 0.236; 0.287 | <0.001 | -- | -0.045 | -.074; -0.016 | 0.002 | --- |
| ***“Health Conscious” dietary pattern score*** | 5013 (100.0) | -0.113 | -0.138; -0.088 | <0.001 | -- | 0.165 | 0.138; 0.192 | <0.001 | --- |
| ***“Traditional/White” dietary pattern score*** | 5013 (100.0) | 0.234 | 0.209; 0.259 | <0.001 | -- | 0.096 | 0.068; 0.124 | <0.001 | --- |
| ***“Fusion” dietary pattern score*** | 5013 (100.0) | 0.024 | -0.002; 0.050 | 0.073 | -- | 0.170 | 0.142; 0.198 | <0.001 | --- |
|  |  | **Maternal characteristics at the 54-month interview** | | | | | | | |
| ***Neighbourhood deprivation (NZDep2013)***  1-3 Least deprived  4-7  8-10 Most deprived | 1744 (30.7)  2044 (36.0)  1888 (33.3) | *Ref.*  0.085  0.494 | 0.029; 0.142  0.437; 0.552 | 0.003  <0.001 | <0.001 | *Ref.*  0.014  0.016 | -0.047; 0.075  -0.046; 0.078 | 0.645  0.617 | 0.410 |
| ***Currently smoking at least 1 cigarette/day***  No  Yes | 5212 (86.9)  789 (13.1) | *Ref.*  0.682 | 0.614; 0.751 | <0.001 | <0.001 | *Ref.*  0.043 | -0.032; 0.177 | 0.265 | 0.265 |

Unadjusted.β, unadjusted beta-coefficient; CI: confidence interval, NCEA, National Certificate of Educational Achievement, NZDep2006, Ref: category of reference, neighbourhood deprivation index 2006, BMI: body mass index.

Missing-models at 54 months (n): Child’s sex (28); child age at the 54-month interview (0) ; maternal education (61); maternal ethnicity (54); maternal age (47); neighbourhood deprivation index (334); body mass index (703); maternal

smoking patterns (^¶^); physical activity before/during pregnancy (597); maternal dietary patterns Junk, Health Conscious, Traditional/White and Fusion (997).

*t-test,** F-test

† Average increase or decrease in the dietary patterns loading scores in relation to the categories of reference.

^¶^As per *Growing up in New Zealand* study anonymity requirement, “<10” represents greater than zero and less than 10 children in the cell.
